# Effectiveness of Metaverse Space–Based Exercise Video Distribution in Young Adults: Randomized Controlled Trial

**DOI:** 10.2196/46397

**Published:** 2024-01-16

**Authors:** Rami Mizuta, Noriaki Maeda, Tsubasa Tashiro, Yuta Suzuki, Sayo Kuroda, Ayano Ishida, Sakura Oda, Tomoya Watanabe, Yuki Tamura, Makoto Komiya, Yukio Urabe

**Affiliations:** 1 Department of Sport Rehabilitation Graduate School of Biomedical and Health Sciences Hiroshima University Hiroshima Japan; 2 Department of Physical Therapy, Faculty of Rehabilitation Kyushu Nutrition Welfare University Fukuoka Japan

**Keywords:** exercise video distribution, exercise, metaverse, physical activity, web-based intervention

## Abstract

**Background:**

In response to the serious lack of physical activity among young adults, recent attempts have been made to encourage young people to exercise through exercise video distribution. However, merely distributing videos does not lead to improved physical activity levels. Metaverse space, which enables web-based interaction through avatars, allows users to watch exercise videos in the same space as other avatars.

**Objective:**

This study explored whether exercise video distribution using metaverse space is effective in improving physical activity levels, along with mental health and locomotive function, among young people.

**Methods:**

In this parallel-group randomized controlled trial participants were recruited using printed poster displays. A total of 48 young adults aged between 18 and 30 years were assigned to 3 groups of 16 each: the metaverse, YouTube, and control group. To encourage exercise, the metaverse group was given an exercise video each week with a load of around 4-8 metabolic equivalents of tasks (METs) for 8 videos delivered in the metaverse space. The YouTube group was sent a URL on YouTube every week to view exercise videos with the same content as the metaverse group. The control group was given no special instructions. The intervention period was 8 weeks. Pre- and postintervention physical activity, well-being, locomotive syndrome risk tests, and social capital were measured. Although this study was not blinded to the participants, the measurers did not know to which group the participants belonged. Mixed model repeated-measures analyses and a post hoc Wilcoxon signed rank sum test were performed to detect the effects of the intervention in all groups.

**Results:**

The results of the mixed model repeated-measures analyses showed a significant interaction between groups and before and after the intervention for total physical activity (metaverse group: pre 737.1, SD 609.5 METs/week, post 1575.4, SD 1071.8 METs/week; YouTube group: pre 661.7, SD 710.7 METs/week, post 911.9, SD 1103.3 METs/week; and control group: pre 930.6, SD 665.1 METs/week, post 844.7, SD 701.8 METs/week; *P*=.04) but none for the indicators of well-being (*P*=.40), locomotive function scale (*P*=.17), and social capital (*P*=.23). A post hoc test showed a significant increase in physical activity in the metaverse group before and after the intervention (*P*=.006).

**Conclusions:**

This study is the first to show that delivering exercise videos through metaverse space can increase physical activity in young adults by providing a gathering space for individuals similarly motivated for exercise practice. This way, the sense of isolation during exercise is reduced compared with merely distributing videos on YouTube. The use of metaverse space in health promotion is likely to spread, and this study provides a useful reference for its exploration.

**Trial Registration:**

ClinicalTrials.gov NCT06019156; https://ichgcp.net/clinical-trials-registry/NCT06019156

## Introduction

Physical inactivity among young people is a serious problem, with 40.3% of men and 66.1% of women in their 20s reporting not exercising even once a week, according to the National Health and Nutrition Survey, 2019 [1]. Surprisingly, a study of 100 college students aged between 18 and 23 years found that 65% of them were in the high-risk group for locomotive syndrome, a condition that reduces physical function and mobility such as standing and walking. A study of young Japanese adults aged between 18 and 20 years revealed that 45.9% of them had a prevalence of frailty-prefrailty [2]. As Japan’s aging society progresses, the number of people requiring nursing care also increases, and the reasons for this need are often locomotive disorders such as falls and joint diseases [3,4]. Therefore, establishing exercise habits at a young age and maintaining and improving locomotive function are important.

The spread of COVID-19 has led to a lack of exercise and a change to a more sedentary lifestyle, which has focused attention on exercise interventions over home-based web-based video delivery methods. This promotion of physical activity through exercise videos was intended to help address people’s lack of motivation to engage in physical activity when gymnasiums and recreation centers had to close and physical distance was required [5]. An exercise intervention study using YouTube reported that when a new exercise video of approximately 5 minutes was delivered once a week for 12 weeks, physical activity and frequency of muscle training increased in the exercise intervention group [6]. Hence, the distribution of exercise videos through YouTube, the most accessible tool for young people, has shown some effectiveness and has become commonplace even as of 2023, when COVID-19–related restrictions have eased.

However, this conventional promotion of physical activity through the distribution of exercise videos has elements that fail to encourage behavioral changes. Self-determination theory, as it relates to exercise implementation, begins with the fulfillment of 3 basic human psychological needs: competency (the experience of achievement and efficacy), autonomy (the experience of motivation), and relatedness (the experience of connection with others) [7]. However, traditional exercise video distribution using YouTube and other media has focused on individual video-watching, which fails to provide opportunities for connecting with other people. In fact, another study of an exercise intervention using YouTube reported improvements in mental health with no increase in physical activities [8]. Studies showed connection with others as an important factor in exercise implementation and that being in an exercise group was more effective in increasing physical activity than exercising alone [9,10]. Further, social encouragement by those around an individual and exercising together had a positive impact on the acquisition of exercise habits [11,12]. In addition, people who shared their step count information using a special app tended to exercise the next day when other people “liked” or commented on their step counts [13]. Moreover, the group that was provided with peer interaction and social support showed positive effects on locomotive functions, such as improved results in stand-up tests, compared with the group that lacked these supportive activities [14]. Thus, adding an element of social community when promoting exercise is more effective.

The metaverse is an internet-based 3D virtual world. For the generation of digital natives born in the 1990s and 2000s, it is expected that the metaverse will increasingly become a space where they spend part of their daily lives [15]. In recent years, the metaverse space has gained attention in the health care field [16-18], and a bibliometric analysis of virtual and augmented reality showed that the metaverse has been adapted for diagnosis, surgical treatment, and rehabilitation of pain, stroke, anxiety, depression, fear, cancer, and neurodegenerative diseases, with satisfactory results [16]. In a metaverse space, people can interact with and encourage others on the web through their avatars, which are their own alter egos, and create a community of people who gather in the same space. Social distance is known to interfere with mental health [19], and the metaverse’s ability to recreate a space for social interaction is expected to help improve the treatment of mental health symptoms such as anxiety, stress, and eating disorders [20-23]. Therefore, the use of the metaverse has the potential to improve mental health. Moreover, encouraging physical activity within the metaverse space has the potential to promote behavioral responses from users, such as motivating people to adopt a healthier lifestyle [24]. Therefore, exercise training using the metaverse is expected to develop as a method to promote physical activity among young people [16]; however, there is insufficient knowledge on whether physical activity can be promoted by distributing exercise videos in the metaverse space. This study verified whether exercise video distribution using an app that provides metaverse space is effective in improving physical activity levels, mental health, and locomotive function among young people. We hypothesized that the distribution of exercise videos in the metaverse space would increase physical activity and have positive effects related to mental health and locomotive function.

## Methods

### Study Design and Participants

This study was a parallel-group, randomized controlled trial. The participants were recruited among Hiroshima University students between August 10 and September 9, 2022. For recruitment, we displayed a poster explaining the study and indicated a contact person available for questions for those considering participation. Several people expressed their willingness to participate. Participants were offered an incentive of US $6.93. Written consent was obtained after we explained the study details face-to-face. All data were collected in person.

This study’s specific participation criteria were as follows: individuals (1) aged 18 and 30 years at the time of obtaining consent, (2) who owned a smartphone or computer and had internet access, and (3) who did not belong to an exercise community such as a sports club. The exclusion criteria were as follows: individuals (1) who had a history of a disease that prohibited exercise, (2) whose physical activity was >3000 metabolic equivalents of tasks (METs)/week because a person with >3000 METs/week is defined as active [25-27], and (3) who were (or possibly) pregnant.

The sample size was calculated using G*power 3.1.9.2 (version 3.1.9.7; Heinrich-Heine-University Düsseldorf). When the effect size was calculated using raw data from a previous study with a similar design, which used a web-based exercise intervention with a physical activity measure as the main outcome [28], a large effect size of 0.34 was obtained with a partial η^2^ of 0.108. When setting α error probability to .05, power (1–β error probability) to .8, number of groups to 3, and number of measurements to 2, the required sample size was 27 participants, with 9 participants per group. A total of 48 participants were required when the dropout rate was approximately 40% [8].

After completion of the baseline prequestionnaire and measurements, 3 participants who met the exclusion criteria and were physically active for >3000 METs/week were excluded. The participants were divided into 3 groups: “metaverse space-based exercise video distribution group (metaverse group),” “YouTube-based exercise video distribution group (YouTube group),” and “no video distribution group (control group)” in a 1:1:1 ratio. The allocation was performed by segregating blocks of size 3 to generate the sequence [29], and the allocation order was hidden until after each group was allocated. A total of 3 separate staff members performed each of the 3 tasks: generating the random allocation sequence, enrolling participants, and assigning participants to the interventions. We explained to all participants the nature of the 3 groups while obtaining their consent. After allocation, the metaverse and YouTube groups received an 8-week intervention in which the exercise videos were distributed, and a control group was established. The intervention period was from October 3 to November 27, 2022. Thereafter, a postquestionnaire survey and measurements were conducted for all 3 groups.

### Intervention

#### Procedure

The recruitment ran for approximately a month, from August 10 to September 9, 2022, and the premeasurements were taken during a 2-week period from September 12 to 25, 2022. The 8-week intervention was conducted from October 3 to November 27, 2022. The postmeasurements were conducted over a period of 1 week (from December 5 to 11, 2022).

#### Metaverse Group

**Figure 1:**
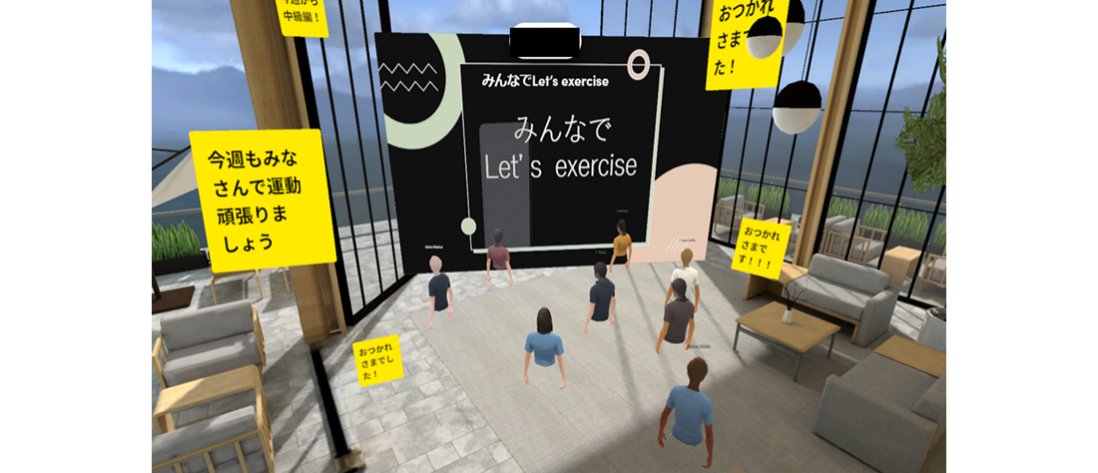
The metaverse space in this study.

The participants in the metaverse group had access to Metaverse space (Spatial Systems Inc), where they could watch exercise videos whenever they wanted (Figure 1). The researcher created a metaverse space for watching exercise videos and set up new videos each week. The URL to access them was shared only with participants in the metaverse group. The unique feature of the metaverse was that users could enter a room to watch exercise videos with their avatars, which were their own alter egos and other avatars. The participants watched exercise videos, which were created by a physical therapist to increase their physical activity, for approximately 5 minutes [6,8]. Then, we instructed them to do the exercise while watching videos. The load setting was approximately 4-8 METs to train the trunk and the upper and lower extremities. A new video was released once a week [6], and the participants could watch 8 videos for 8 weeks. They were informed in advance that new videos would be updated every Monday and, as a reminder, were sent a SMS text message when a new video was available. A web-based survey regarding exercise video viewing frequency was administered, and adherence was tracked weekly.

#### YouTube Group

The YouTube group was sent a message with the URL to access the exercise video every Monday. The YouTube group could watch videos at their preferred times by clicking on a link. The content and time of the video were identical to those of the metaverse group. As in the metaverse group, adherence to exercise implementation was confirmed through a web-based questionnaire, and video update reminders were sent every week.

#### Control Group

Participants were informed by email that they were assigned to the control group simultaneously with the start of the intervention in the Metaverse and YouTube groups. We asked the participants in the control group to spend 8 weeks as usual, without any special instructions or delivery of videos. This group was established as an indicator of the amount of physical activity that may be significantly affected by the infection status and government directives during the COVID-19 pandemic.

### Outcome Measurements

#### Basic Information

The participants were asked and tested for age, height, weight, BMI, lifestyle, sex, living status, and frailty to characterize the basic information. The weight and frailty tests were measured by a physical therapist, and the other parameters were measured using a questionnaire.

#### Lifestyle

Breslow’s 7 health practice scores were used to assess lifestyle [30,31]. The 7 parameters were: smoking (presence or absence), alcohol intake (high or low: consumption of ≥5 or <5 alcoholic beverages), sleep (≥7 hours or <7 hours), breakfast (eating or not eating), eating between meals (eating or not eating), exercise (≥2 times, 30 minutes/week or <2 times, 30 minutes/week), and proper weight (BMI <25 kg/m^2^ or ≥25 kg/m^2^).

#### Living Status

Regarding their living status, the participants were asked whether they lived alone or with others.

#### Frailty Test

The Japanese Cardiovascular Health Study criteria questionnaire was designed to indicate potential frailty, and its contents are as follows [32,33]: (1) have you lost 2 kg or more in the past 6 months? (2) Do you engage in moderate levels of physical exercise or sports aimed at improving health? (3) In the past 2 weeks, have you felt tired without reason? (4) Hand grip strength of <26 kg in men or <18 kg in women, and (5) gait speed of <1 m/second. Grip strength was measured using a hand dynamometer (TKK 5401; Takei). Each participant performed 2 trials, and the best value from both trials was used for the analysis. Walking speed was calculated by performing a 10-meter walk test and dividing the measured value by 10 to obtain the results per meter. All questions were rated 1 or 0, and higher scores indicated a higher possibility of frailty. Frailty, prefrailty, and robustness were defined by the number of applicable items: 3-5, 1-2, and 0.

### Primary and Secondary Outcomes

The study assessed physical activity, well-being, locomotive syndrome risk tests, and social capital before and after the intervention for 8 weeks.

#### Primary Outcome: Physical Activity

The short form of the International Physical Activity Questionnaire was used to measure the effect of an intervention using exercise videos with different distribution methods (Metaverse and YouTube) on physical activity [34]. Total physical activity was measured as the average amount of vigorous physical activity, the amount of moderate activity, and the amount of walking (METs minutes/week). A MET is defined as the amount of energy required for a person to sit quietly. In the premeasurement, the participants answered for the most recent week, and in the postmeasurement, they responded for the week after the intervention was completed.

#### Secondary Outcomes: Well-being, Locomotive Syndrome Risk Tests, and Social Capital

##### Well-Being

To assess well-being and mental health, we used the Japanese version of the World Health Organization-Five Well-being Index [35]. Responses to each item were rated on a 6-point scale from 0 to 5 and consisted of 5 questions. The highest score is 25, with higher scores indicating better well-being.

##### Locomotive Syndrome Risk Test

We used the 25-question geriatric locomotive function scale, proposed by the Japanese Orthopedic Association [33,36], to measure levels of locomotive function based on normal daily activities and pain. All the items were answered on a scale of 0-4. The higher the score, the more impaired the motor function, which indicates a decline in mobility and interference with social life.

##### Social Capital

The social capital questions consisted of 11 items related to civic participation, social cohesion, and reciprocity, with a perfect score of 11 and higher scores indicating stronger social connections [37]. We included this question item because of the possibility that the sense of social participation could change in the metaverse group.

#### Supplemental Outcomes: Impression of Each Intervention (Metaverse and YouTube Groups)

We interviewed each of the metaverse and YouTube groups regarding their impressions of the intervention using exercise videos after the 8-week intervention. User experience is the experience of a user with the use of a product, system, or service. From the review of user experience, we selected the following list of questions: novelty, relatedness (connection with others), motivation, excitement, satisfaction, delight, comfort, attractiveness, expectation, and fulfillment [38]. The participants answered each question with a “good,” “rather good,” “rather poor,” or “poor” response.

### Statistical Analysis

Primary and secondary outcomes were analyzed by performing a mixed model repeated-measures ANOVA. This analysis was used to detect the effect of interventions between the metaverse, YouTube, and control groups. We assessed the main effect as well as group and time interactions on the outcome measure. A mixed model repeated-measures analysis is an intention-to-treat analysis with unbiased estimates that considers all available data from participants enrolled in the study [39]. The effect size *r* for the interaction effect of a mixed model repeated-measures analysis was calculated using F statistics. As a posttest, the Wilcoxon signed rank sum test was performed after confirming nonnormality to compare the pre- and postintervention outcomes of each group. The z-statistic was used to calculate the effect size *r*. Supplemental outcomes were analyzed by performing chi-square tests to compare the intervention impressions between the metaverse and YouTube groups. Note that to use the chi-square test, “rather good” was included in “good” and “rather poor” was included in “poor,” thus reorganizing responses into 2 groups. The effect size was calculated using Cramer *V*.

Statistical analyses were performed using SPSS (version 28.0; IBM Corp). The significance level was set at 5%.

### Ethical Considerations

This study was approved by the Ethical Committee for Clinical Research at Hiroshima University (C2022-0004) and registered with UMIN (UMIN000048046). We followed the guidelines of the Consolidated Standards for Reporting Trials [40]. There were no significant changes in the method used after the study’s initiation.

## Results

### Participants

**Figure 2:**
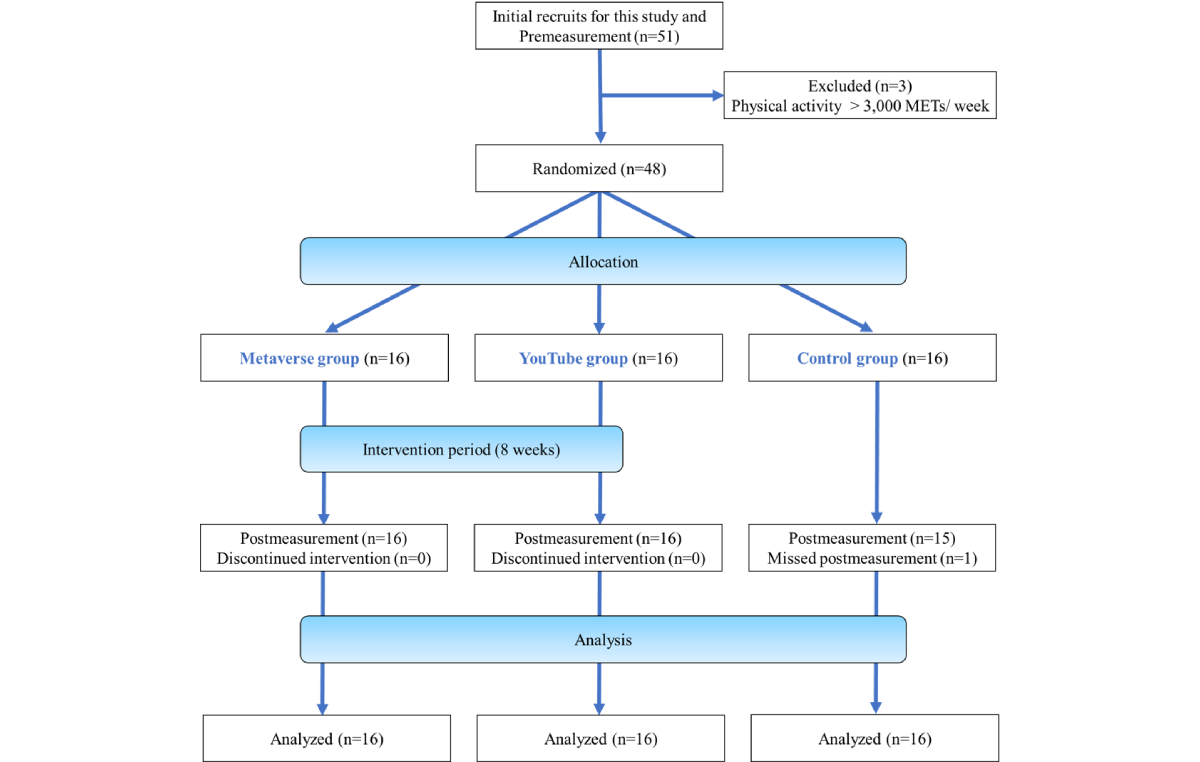
Study flowchart. MET: metabolic equivalents of task.

Figure 2 shows the participant flowchart. A total of 51 individuals expressed interest in the study; however, 3 were excluded because their physical activity was >3000 METs/week. All the participants in the metaverse and YouTube groups completed the intervention. Participants were also interviewed weekly during the 8-week intervention period to monitor their exercise implementation. The average frequency of exercise while watching the exercise video per week was 4.1 (SD 3.9) and 2.6 (SD 1.6) for the metaverse and YouTube groups, respectively (response rates: metaverse group 100% and YouTube group 99%). For the postmeasurement, a participant in the control group could not participate owing to illness.

### Basic information, Lifestyle, Living Status, and Frailty Test

Table 1 presents the participants’ demographic characteristics (mean 22.4, SD 2.4 years; 32/48, 67% women). Prefrailty accounted for 41/48 (85%) participants because we recruited those who were not members of a sports club and whose physical activity was <3000 METs/week.

**Table 1:** Participant’s demographics at baseline.

Variables			Total		Metaverse group (n=16)		YouTube group (n=16)		Control group (n=16)
Age (years), mean (SD)			22.4 (2.4)		22.9 (1.4)		22.5 (2.0)		22.0 (3.4)
Height (cm), mean (SD)			164.0 (7.9)		162.4 (6.6)		163.9 (6.2)		165.7 (10.3)
Weight (kg), mean (SD)			57.7 (11.6)		56.7 (9.8)		57.3 (10.2)		59.0 (14.8)
BMI (kg/m²), mean (SD)			21.3 (2.9)		21.4 (2.5)		21.2 (2.5)		21.3 (3.6)
Lifestyle, mean (SD)			4.6 (0.9)		4.6 (0.9)		4.4 (0.7)		4.7 (1.1)
**Sex, n (%)**									
	Male	16 (33)		6 (38)		6 (38)		5 (31)	
	Female	32 (67)		10 (63)		10 (63)		11 (69)	
**Living status, n (%)**									
	Alone	31 (65)		11 (69)		10 (63)		10 (63)	
	With others	17 (35)		5 (31)		6 (38)		6 (38)	
**Frailty test, n (%)**									
	Frailty	2 (4)		1 (6)		1 (6)		0 (0)	
	Prefrailty	41 (85)		13 (81)		14 (88)		14 (88)	
	Robust	5 (10)		2 (13)		1 (6)		2 (13)	

### Physical Activity, Well-Being, Locomotive Syndrome Risk Test, and Social Capital

Table 2 shows the estimation results of the fixed effects from the mixed model repeated-measure analysis. Only total physical activity showed a significant interaction (*F*_2, 45.042_=3.338, *P*=.04; effect size=0.263). The main effects for the time showed a significant difference in vigorous activity (*F*_1, 45.083_=6.921, *P*=.01), locomotive function scale (*F*_1, 44.895_=9.557, *P*=.003), and social capital (*F*_1, 44.650_=5.085, *P*=.03).

Table 3 shows the results of the post hoc test comparison of the difference in total physical activity before and after the intervention in each group. In the metaverse group, a significant difference was detected between pre- and postintervention periods (*P*=.006; effect size=0.682).

**Table 2:** Estimate of the fixed effects from the mixed model repeated-measures analysis, (N=16).

Variables							Metaverse group		YouTube group		Control group		Main effect												Interaction effect						
													Time					Group						Time*Group							
													*F* test (*df*)		*P* value			*F* test (*df*)			*P* value			*F* test (*df*)				*P* value		Effect size	
**Physical activity: total (METs^a^ minutes/week), mean (SD)**														5.153 (1, 45.051)		.03			1.383 (2, 45.297)			.26				3.338 (2, 45.042)			.04		0.263
	Pre						737.1 (609.5)	661.7 (710.7)		930.6 (665.1)																					
	Post						1575.4 (1071.8)	911.9 (1103.3)		844.7 (701.8)																					
**Physical activity: vigorous (METs minutes/week), mean (SD)**														6.921 (1, 45.083)		.01			3.140 (2, 45.257)			.053				1.917 (2, 45.075)			.16		0.202
		Pre					237.5 (423.4)	120 (231.9)		137.5 (253.8)																					
		Post					745 (677.4)	345 (613.90)		162.7 (493.3)																					
**Physical activity: moderate (METs minutes/week)**														2.004 (1, 44.498)		.16			0.994 (2, 44.623)			.38				0.748 (2, 44.490)			.48		0.129
			Pre				211 (270.3)	162.5 (319.2)		311.3 (458.2)																					
			Post				425 (347.1)	235 (411.5)		317.6 (351.9)																					
**Physical activity: walking (METs minutes/week)**														0.013 (1, 45.119)		.91			0.192 (2, 45.253)			.83				1.488 (2, 45.110)			.24		0.179
					Pre		288.6 (255.8)	379.2 (441.9)		481.8 (552.1)																					
					Post		636.5 (686.8)	331.9 (368.1)		364.4 (284.0)																					
**Well-being**														2.449 (1, 44.726)		.12			1.189 (2, 45.186)			.31				0.934 (2, 44.718)			.40		0.143
						Pre	15.4 (3.9)		16.4 (4.1)		18.4 (4.1)																				
						Post	17.6 (5.5)		17.3 (3.9)		18.5 (4.8)																				
**Locomotive function scale**														9.557 (1, 44.895)		.003			0.429 (2, 45.308)			.65				1.821 (2, 44.887)			.17		0.197
				Pre			3.9 (3.4)	3.8 (3.3)		3.5 (3.7)																					
				Post			1.1 (1.9)	2.6 (2.9)		2.9 (3.3)																					
**Social capital**														5.085 (1, 44.650)		.03			2.348 (2, 45.142)			.11				1.519 (2, 44.642)			.23		0.181
			Pre				5.9 (1.9)	4.9 (1.8)		6.2 (1.3)																					
			Post				7.1 (2.4)	5.7 (2.0)		6.2 (1.5)																					

^a^MET: metabolic equivalents of task.

**Table 3:** Differences between pre- and postintervention outcome measures (N=48).

Variables			Physical activity: total (metabolic equivalents of tasks minutes/week)
**Metaverse group**			
	Pre, mean (SD)	737.1 (609.5)	
	Post, mean (SD)	1575.4 (1071.8)	
	*P* value	.006	
	Effect size	0.682	
**YouTube group**			
	Pre, mean (SD)	661.7 (710.7)	
	Post, mean (SD)	911.9 (1103.3)	
	*P* value	.23	
	Effect size	0.298	
**Control group**			
	Pre, mean (SD)	930.6 (665.1)	
	Post, mean (SD)	844.7 (701.8)	
	*P* value	.57	
	Effect size	–0.142	

### Comparison of Impressions of the Intervention Between the Metaverse and YouTube Groups

**Figure 3:**
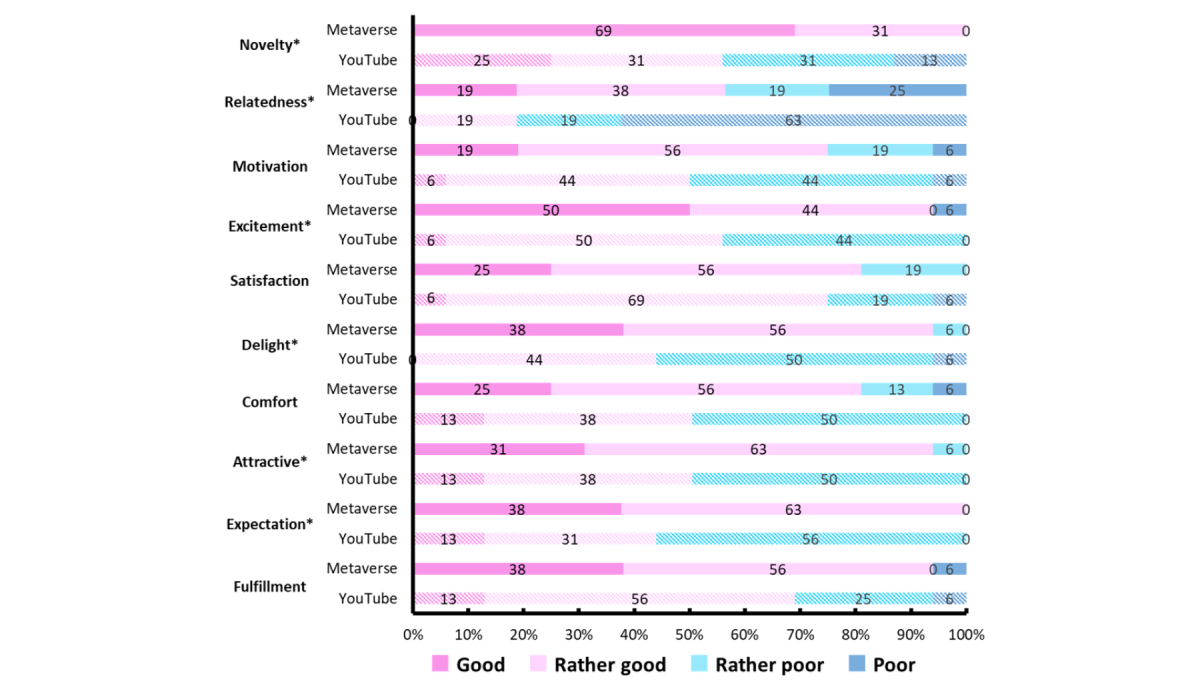
Impressions of intervention in the Metaverse and YouTube groups. *P<.05.

Multimedia Appendix 1 and Figure 3 show the results of a chi-square test on impressions of the intervention between the metaverse and YouTube groups. Significant differences were observed between the 2 groups in novelty (*P*=.003; effect size=0.529), relatedness (*P*=.03; effect size=0.387), excitement (*P*=.01; effect size=0.433), delight (*P*=.002; effect size=0.539), attractiveness (*P*=.006; effect size=0.487), and expectation (*P*<.001; effect size=0.626). The results did not reveal significant differences in motivation, satisfaction, comfort, or fulfillment.

## Discussion

This study examined whether an exercise intervention using exercise videos in the metaverse space has a positive impact on physical activity. The main results show a significant interaction between groups and time (pre- and postintervention) in total physical activity, with post hoc analysis showing a significant increase in total physical activity in the metaverse group after compared with before the intervention and no significant change in the YouTube and control groups. This study is the first to show that using the metaverse space to deliver exercise videos can promote increased physical activity.

The current results support the hypothesis that the use of the metaverse increases physical activity. The exercise videos used in the metaverse, and the YouTube groups are identical, and the metaverse group differs from the YouTube group in that the exercise videos can be viewed in a shared space with other avatars in the metaverse space. Studies have noted that interacting with others is more effective than exercising alone at increasing physical activity and sustaining exercise [9-13]. Metaverse characteristics include the ability to form communities within virtual reality and the persistence of virtual reality regardless of individual user access [15]. In this study, no change in social capital was detected before and after the intervention; however, the percentage of those who had an impression of relatedness (connection with others) was also significantly higher in the metaverse group (9/16, 56%) than in the YouTube group (3/16, 19%)—indicating that more people felt connected to others when using the metaverse than when using YouTube. Additionally, strategies to increase physical activity include improving the physical environment, such as its construction, and the social environment, including social support [41]. The use of the metaverse resulted in the persistence of a purposeful social space for exercise that could be accessed at any time. Therefore, the existence of the metaverse space is an aspect of environmental improvement for exercise for participants who previously had no exercise habits, leading to increased exercise implementation and physical activity.

The reason indicators other than physical activity did not improve in the metaverse group is also an important area to consider. This study asked about well-being as an item that could be related to mental health. Exercise is known to promote the secretion of estradiol and serotonin and regulate cortisol levels, an internal change that reduces depression and other depressive symptoms [42-44]. Additionally, reducing loneliness and encouraging social interaction decreases the risk of depression and mental health problems [45]. Therefore, we expected to observe mental health improvements in the metaverse group; however, there were no significant differences among the 3 groups. The standard for suspicion of mental problems is a cutoff value of ≤11 points for well-being [35,46]. However, the mean values for participant well-being did not correspond to the cutoff values. Thus, mental health changes were unlikely to be observed even if the amount of physical activity increased because the group did not initially have low well-being.

The locomotive function scale, a measure of body pain and locomotor function, showed an improving trend in the metaverse group; however, the difference was not significant. A previous study found pain relief after an 8-week exercise program, including strength training of the trunk, in participants with low back pain that appeared to be caused by inactivity [47]. Several studies have reported muscle hypertrophy occurring within 6-8 weeks [48]. These facts indicate that if training is managed meticulously in the right way, the body can be changed in a period of 8 weeks. However, it is possible that the unmonitored, 8-week intervention period did not result in sufficient changes in motor function, even though the amount of physical activity increased. To improve motor function, training to improve muscle strength and endurance should be continued at least 3 times per week [49].

As for the differences in impressions of the intervention between the metaverse and YouTube groups, many factors such as novelty, relatedness, excitement, delight, attractiveness, and expectation were more positive in the metaverse group than in the YouTube group. Regarding novelty, a previous study stated that sufficient novelty for the target audience leads to successful health support using the app [50]. Even for young adults who were relatively familiar with and used apps and social networking services on a regular basis, the metaverse seemed to be a novelty, which may have supported the implementation of the exercise in this study. Delight is an important factor in many areas of life [51]. Studies have reported that delight is the most important predictor of commitment and participation in general youth and elite sports [52,53]. This amplification of delight using a metaverse space to distribute videos is meaningful in promoting exercise. Interestingly, the metaverse group found the intervention more attractive. A previous study that used apps to promote healthy lifestyles, including regular exercise, found that a combination of different services, such as health-related quizzes and the ability to record health status, led to successful interventions [54]. Exercise video-delivered studies have also shown that simply delivering videos does not increase physical activity [8]. The essence of using the metaverse may have increased the attractiveness of exercise video distribution in this study.

This study examined the effectiveness of exercise video distribution using metaverse space by comparing the previously used YouTube and a control population. This study’s strength lies in making the first attempt to distribute exercise videos using metaverse space. Furthermore, it shows the potential of using metaverse space to improve the physical activity level of young adults.

However, this study has some limitations. First, selection bias may have occurred. We recruited participants who were not members of an athletic club and who did not exercise heavily. However, it is possible that the target population was composed of students who were interested in exercising, as suggested by their interest in participating in this study. Additionally, we recruited participants from a single university. The risk of selection bias may be reduced by conducting a study on a larger scale and involving more institutions. However, the 3 groups were randomly assigned in this study, and the required, precalculated sample size was secured. Second, it was difficult to accurately determine the time and frequency of interactions between participants in the metaverse space. If a system can be implemented to monitor the time of entry into the metaverse space, the frequency of interactions, and so on, the mechanism for promoting physical activity through the use of the metaverse space could be made more visible. Third, participants could receive the intervention at the time of their choosing; however, the start time of the intervention was not monitored. The effectiveness of the intervention could be moderated or altered by the time of the intervention, and future researchers should accurately monitor the start time of the intervention. We note that the main outcome, the calculation of physical activity, did not depend on the starting time of the intervention. Final, there was room for improvement in the comfort of the interventions in the metaverse group. This study used metaverse space in the manner in which the videos were arranged. Metaverse space (Spatial, Spatial Systems, Inc) has the advantage of being a service that anyone can use free of charge; however, if industry and academia collaborate to devise a system more suited to the distribution of exercise videos and the promotion of exercise, it could have a more positive effect on physical activity and other aspects.

In the near future, services allowing access to the metaverse will likely continue to expand. Therefore, the number of opportunities for the general public to experience the metaverse will further increase. The fact that 100% of the metaverse group in this study had high expectations is a hopeful part of the development of using the metaverse to improve physical activity. This research provides a basis for establishing further methods of using the metaverse to promote exercise.

## Appendix

Multimedia Appendix 1 Comparison of impressions on intervention between the metaverse and YouTube groups.

Multimedia Appendix 2 CONSORT-eHealth (V 1.6.1).

## Abbreviations

MET: metabolic equivalents of task
