# Supplementary material for: Effectiveness of Metaverse Space–Based Exercise Video Distribution in Young Adults: Randomized Controlled Trial
Source: JMIR Mhealth Uhealth. 2024 Jan 16;12:e46397. doi: 10.2196/46397 (PMC10828949; doi:10.2196/46397)
Supplement: Multimedia Appendix 1 [file mhealth_v12i1e46397_app1.pdf]

Variables	Score	Total (n = 32)	Metaverse group (n = 16)	YouTube group (n = 16)	χ^2	*P*	Effect size
Novelty	Good	25 (78)	16 (100)	9 (56)	8.960	.003	0.529
	Poor	7 (22)	0 (0)	7 (44)			
Relatedness	Good	12 (38)	9 (56)	3 (19)	4.800	.03	0.387
	Poor	20 (63)	7 (44)	13 (81)			
Motivation	Good	20 (63)	12 (75)	8 (50)	2.133	.14	0.258
	Poor	12 (38)	4 (25)	8 (50)			
Excitement	Good	24 (75)	15 (94)	9 (56)	6.000	.01	0.433
	Poor	8 (25)	1 (6)	7 (44)			
Satisfaction	Good	25 (78)	13 (81)	12 (75)	0.183	.70	0.076
	Poor	7 (22)	3 (19)	4 (25)			
Delight	Good	22 (69)	15 (94)	7 (44)	9.309	.002	0.539
	Poor	10 (31)	1 (6)	9 (56)			
Comfort	Good	21 (66)	13 (81)	8 (50)	3.463	.06	0.329
	Poor	11 (34)	3 (19)	8 (50)			
Attractive	Good	23 (72)	15 (94)	8 (50)	7.575	.006	0.487
	Poor	9 (28)	1 (6)	8 (50)			
Expectation	Good	23 (72)	16 (100)	7 (44)	12.522	< .001	0.626
	Poor	9 (28)	0 (0)	9 (56)			
Fulfillment	Good	26 (81)	15 (94)	11 (69)	3.282	.07	0.320
	Poor	6 (19)	1 (6)	5 (31)			

Data are expressed as n (%).
